# Occupational Health Hazards in ICU Nursing Staff

**DOI:** 10.1155/2010/849169

**Published:** 2011-02-07

**Authors:** Helena Eri Shimizu, Djalma Ticiani Couto, Edgar Merchán-Hamann, Anadergh Barbosa Branco

**Affiliations:** ^1^Department of Nursing, University of Brasilia, SQN 205- G-605, 70843-070 Brasilia, DF, Brazil; ^2^Nursing Division, Hospital de Base of the Federal District, 71917-000 Brasilia, DF, Brazil; ^3^Department of Collective Health, University of Brasilia, 70910-900 Brasilia, DF, Brazil

## Abstract

This study analyzed occupational health hazards for Intensive Care Unit (ICU) nurses and nursing technicians, comparing differences in the number and types of hazards which occur at the beginning and end of their careers. A descriptive cross-sectional study was carried out with 26 nurses and 96 nursing technicians from a public hospital in the Federal District, Brazil. A Likert-type work-related symptom scale (WRSS) was used to evaluate the presence of physical, psychological, and social risks. Data were analyzed with the use of the SPSS, version 12.0, and the Kruskal-Wallis test for statistical significance and differences in occupational health hazards at the beginning and at the end of the workers' careers. As a workplace, ICUs can cause work health hazards, mostly physical, to nurses and nursing technicians due to the frequent use of physical energy and strength to provide care, while psychological and social hazards occur to a lesser degree.

## 1. Introduction

It is increasingly recognized that health workers, especially nurses and nursing technicians, are subject to a variety of health hazards. Several studies have indicated the need to identify, in a critical area such as an ICU, the factors causing hazards, as well as strategies to avoid them, so that the health of these workers is not affected [1, 2]. Signs of strain in nurses and nursing technicians are manifested in various forms, depending on the task complexity. Certainly, one of the most common strains is physical and mental fatigue.

In Brazil, the nursing staff at an ICU is generally comprised of two technically distinct occupations: nurses and nursing technicians. Nurses are primarily involved in organizing the environment, material resources, and coordinating the work of the nursing staff, as well as providing care, principally in more complex procedures. Nursing technicians are generally responsible for direct patient care tasks, that is, hygiene, administering medication, placing and changing bandages, and so forth.

Certain studies have suggested that signs of intense mental fatigue are slightly more frequent in nurses who occupy positions which are hierarchically superior to nursing technicians, possibly due to workloads associated with the position held in the labor process [1, 2]. Other studies demonstrate that nursing technicians are more exposed to excessive physical workloads, due to the physical care required by patients [3].

Despite the technical and hierarchical differences, nurses and nursing technicians are exposed to intense fatigue, given the fundamental roles played in critical patient care. These include rapid decision-making, and a keen sense of responsibility in determining priority actions; the resolution of complex problems; continuous reorganizing of activities as a result of frequent interruptions; management of large volumes of variable data due to simultaneous treatments; a wide variety of interventions required by critical clinical conditions.

The work of an ICU nursing team may thus be characterized by uncertainties, variable situations, and the need for immediate action, requiring high levels of knowledge, skill, and competence, as well as psychomotor, affective and cognitive control—which may generate fatigue.

In addition, the ICU nursing team is almost permanently faced with human suffering and death and thus must deal with ambiguous feelings, not only with regard to patients, but family members as well. This requires workers to remain level-headed, and to use strategies to deal with the psychological burdens produced by the working conditions to offset tensions that may cumulatively affect their health, potentially leading to pathogens [2, 4].

Notwithstanding the essential nature of the work carried out by nurses and nursing technicians in critical environments, various social and historical aspects are also involved, such as insufficient social recognition, as illustrated by the undervalued and invisible nature of this position in relation to other healthcare professionals, including by those seeking assistance [5].

Studies have shown that the direct care of people who are physically ill or disabled, associated with precarious working conditions characterized by long work schedules, low salaries, the need to hold two jobs, and the carrying out of unpleasant tasks, affects mental health, contributing to the occurrence of job accidents, and even the reduction of life expectancy [6–9].

Given the above, and based on the psychodynamics labor theory, it is assumed in this study that work at ICUs causes various types of fatigue, which may reduce satisfaction (pleasure) and effective material and social recognition, leading to dissatisfaction (suffering), exploitation, illness, and even death [10, 11]. Thus, it is extremely important to determine those factors that contribute to fatigue, in order to propose transformations needed to mitigate the effects of work processes in an organization.

However, in spite of the potential health risks resulting from the organizational context, positive aspects of the occupation are being rediscovered as a way of avoiding and overcoming the negative aspects, through a line of argumentation that emphasizes the role played by positive subjective experiences which may improve the lives of nursing staff and avoid pathologies [12–14]. Positive work experiences help enrich individual identities, and to maximize the development of individual potentials.

From this perspective, the dynamic and complex nature of caring for critical patients, which involves interaction between subjects—the care provider and care receiver, demands making choices, arbitration, and prioritizing actions and objectives. Such interaction guides workers' decision-making, enabling them, to a certain extent, to deal with the fragmentation of the work process. Workers thus take responsibility for the management of their work [13, 14] by continuously negotiating between the established norms, routines and procedures, and the sudden demands of daily care. The discovery of new possibilities would then make work more gratifying for workers and result in better care for patients. 

In this sense, this study may help to consider the work processes of ICU nurses and nursing technicians from a different perspective, and the development of action plans aimed at reducing suffering that benefit workers' health, and avoid illness at work. In this context, we analyzed work-related hazards affecting nurses and nursing technicians working at a Brazilian ICU and compared the occurrence of these hazards at the beginning and end of their professional careers.

## 2. Method

This is a descriptive cross-sectional study aimed at determining the prevalence of an event in a given population, in order to provide explanations for the variations in their frequency [15], which was carried out with nurses and nursing technicians at the ICU at the Hospital de Base (HBDF), a public institution located in Brasilia, DF, Brazil. The ICU is comprised of 38 beds: 12 of which are reserved for adult patients; 10 for pediatric patients; 8 for trauma cases; 8 for patients with coronary problems. 

### 2.1. Sample

The ICU staff where the study was conducted has a total of 126 nursing workers. The sample, which was selected in a nonprobabilistic manner, was comprised of 122 participants, 26 of whom were college-educated nurses, responsible for care management [16], and 96 nursing technicians, who are certified technical professionals responsible for direct care of patients, and who work under the supervision of nurses [16]. Excluded were those who refused to sign the term of free and informed consent (*n* = 1), those who were absent from work for more than 90 consecutive days (*n* = 2), and those who had less than six months of specific work experience (*n* = 1).

### 2.2. Data Collection Instruments and Variables

The data collection instrument was a questionnaire, which was used to characterize the socio-demographic profiles of the study's subjects: marital status, level of schooling, and number of jobs. The study also used the work-related symptom scale (WRSS), which is part of the work-related illness risk Inventory (WRIRI) (consisting of a total of four scales), an auxiliary instrument used to diagnose critical work indicators, which was developed and validated by Ferreira and Mendes [17] in a national study carried out jointly with the National Federation of Brazilian Social Security Fiscal Auditors. WRSS is a five-point Likert scale, comprised of 32 items, used to evaluate the presence of hazard-related factors, which attributes a value of 1 to a totally absent symptom, and a value of 5 to a highly present symptom [17]. It is organized into three factor categories: physical hazards, psychological hazards, and social hazards, all resulting from the situations faced in work settings.

The first category, physical hazards, is defined as bodily pain and biological disturbances and is comprised of 12 items, with an **α** = 0.849: sleep disturbances, leg pains, back pains, body pains, circulatory disturbances, arm pains, shifts in appetite, digestive disturbances, visual disturbances, auditory disturbances, and respiratory disturbances. 

The second category, psychological hazards, is defined as negative self-perception, negative outlook on life in general, and shifts in mood and is comprised of 11 items and **α** = 0.92: sadness, irritation with everything, loss of self-confidence, feeling of emptiness, loss of self-control, bitterness, feeling of defeat, crying for no apparent reason, willingness to give everything up, long-lasting feeling of despair, and negative image of oneself. 

Lastly, the third category, social hazards, is defined as a feeling of isolation and difficulties in family relationships, encompassing nine items and **α**= 0.88, family relation difficulties, affective relation difficulties, insensitivity towards others, social life difficulties, difficulty in making friends, social isolation, difficulty in making decisions regarding personal life, overall disinterest towards others, and uncontrolled aggressiveness.

On the WRSS scale, the results were classified on three levels: between 1.0 and 2.3 were considered satisfactory; between 2.3 and 3.7 moderate; higher than 3.7 were considered critical. The WRSS scale was applied to the same individuals when asked to refer to two moments in their lives: when they began their careers (the first seven years) and at the end of their careers (after 15 years of work).

Data were collected between September 2006 and December 2007 by one of the researchers. The time spent on answering the questionnaires varied between 15 to 20 minutes.

### 2.3. Data Analysis

The collected data were analyzed with the Statistical Package for the Social Sciences for Windows (SPSS), version 12.0. The data were then transferred to Microsoft Office XP Excel for formatting.

In the data analysis, the central tendency and dispersion values were calculated based on the data obtained from the WRSS. A Kruskal-Wallis nonparametric test was performed to rank the data according to their significance, based on their mean values, in order to test the null hypothesis, which assumes that the populations are equal with regard to differences in the physical, psychological, and social hazard factor categories present in the WRSS scale. The Kruskal-Wallis test was used because the numeric dependent variable (WRSS scale) was not normally distributed.

The factors detected in nurses and nursing technicians at the beginning of their careers (first seven years) were compared with those at the end of their careers (over 15 years of work), in their respective categories. 

### 2.4. Ethical Aspects Related to the Study

This study was approved by the Research Ethics Committee of the Federal District's Health Secretariat, under registry no. 183/05. All subjects then signed the Free and Informed Consent Term, as set forth by Resolution no. 196/96, of the Ministry of Health.

## 3. Results

With regard to the profile of the study's subjects, the percentage of women was considerably higher (76.9% of nurses, and 84.4% of nursing technicians), confirming the same trend of other areas of nursing. There was also a predominance of workers who were married in both the nurse and nursing technician categories: 53.8% and 53.1%, respectively.

In relation to level of schooling, most nurses had graduate-level education (80.8%), while among nursing technicians, high school education prevailed (54.3%)—which is the minimum required to carry out the function. It must be pointed out, however, that among the latter, there was also strong evidence of undergraduate or graduate study, reaching 37.3%.

In regard to the number of jobs, 65.4% of nurses held two or three jobs, indicating a large work overload; among nursing technicians, most only held one job: 60.4%.

The data in Table 1 indicate that work at ICUs caused moderate physical hazards; however, other health hazards were also detected. The levels of psychological and social hazards were considered satisfactory.

With regard to physical hazards (Table 2), leg pains and sleep disturbance symptoms were at critical levels among nurses; among nursing technicians, critical levels were detected for leg and back pains.

Back pains, body pains, and circulatory disturbances were considered moderate among nurses; among nursing technicians, these included sleep disturbances, body pains, and arm pains.

Among nurses, health risks were manifested by arm pains, shifts in appetite, and visual, digestive, auditory, and respiratory disturbances; among nursing technicians, circulatory, visual, digestive, auditory, and respiratory disturbances were considered at satisfactory levels.

Concerning psychological hazards (Table 3), it may be verified that for both categories, levels were satisfactory with regard to negative feelings (loss of self-confidence, loss of self-control, feeling of emptiness, bitterness, feeling of defeat, crying for no apparent reason, willingness to give everything up, long-lasting feeling of despair, and negative image of oneself). A moderate level of sadness was identified among nurses, being considered satisfactory for nursing technicians.

As shown in Table 4, levels were considered satisfactory for all hazards for both nurses and nursing technicians.

The Kruskal-Wallis test was applied to the scores obtained for nurses and nursing technicians regarding the presence of physical, psychological, and social hazard factors between the beginning and end of their careers (Table 5). With regard to physical hazards, it was verified that for both nurses and nursing technicians, there was a slight decrease in hazard level, which was not statistically significant.

In relation to psychological hazards, the reduction observed between the beginning and end of the careers was more expressive, especially among nursing technicians, but no statistically significant association was detected.

Lastly, with regard to physical hazards, there was a slight decrease among nurses, and a small increase among nursing technicians, also with no statistical significance.

## 4. Discussion

The study found that the levels of physical hazards for nurses and nursing technicians working at ICUs were critical at a single Brazilian institution. Albeit the psychological and social hazard levels were tolerable, they nevertheless indicated the need to introduce changes in both the context and process of the referred line of work.

Concerning physical hazards, it was verified that the most frequent manifestation was pain in some part of the body, due to the amount of physical effort put into patient care, which has not been reduced with the use of technology. Sleep disturbances were also detected, especially among nurses, as a consequence of working two shifts—possibly provoking certain psycho-emotional illnesses [18]. Another aspect to be considered is that most of these professionals held more than one job to maintain family income levels.

Body pain was mostly present in nurses and nursing technicians' legs, since for most procedures, professionals remain standing most of the time (bathing, oral hygiene, moving of patients, feeding, administering medication, placing and removing bandages, collecting biological material, on recumbent patients, who are frequently unconscious, or in coma). In addition, workers spend most of their work shift moving from one place to another, since the required material and equipment is rarely in the vicinity of patients' beds. Also common is the manifestation of circulatory problems, such as varicose veins, edemas, and other problems, in workers with many years of service.

Another type of problem manifested by nurses and nursing technicians at ICUs is dorsal pain. Studies have shown that a great deal of spinal injuries is caused by stereotyped or inadequate body posture as a result of the work carried out [6, 19–21]. Although beds at ICUs are higher to facilitate patient care, workers need to bend over constantly, either to place or change a bandage, to move a patient, or provide other types of care.

Posture problems, which affect a large number of nurses and nursing technicians, possibly causing damage to the muscular-skeletal system, are manifested through some type of pain, frequently aggravated by ambient characteristics, and equipment [3, 6, 19, 22].

Activities involving prolonged static contractions or the immobilization of parts of the body (such as the head, neck, or shoulders), chronic tension, strenuous physical activity, elevation and abduction of the arms above the height of the shoulders, and bodily vibrations may all cause pain—which may irradiate to lower members, and become persistently localized. Cervical pain, which is characterized by the presence of spontaneous pain or by palpations and/or edemas in the cervical region, may also develop [6].

Ergonomic studies have been carried out to analyze the physical postures acquired during nursing activities, in order to adapt them to the principle of biomechanics [6, 19]. These studies indicate the need to invest in workers' education, to increase awareness regarding correct body positioning during the course of their activities, especially in critical areas, such as ICUs [6, 7, 20, 22–25]. However, for certain ergonomists, this is not sufficient. Work processes also need to be modified, so that workers may assume more adequate and comfortable body postures [23, 26].

In addition to physical exhaustion, the present study also detected that work at ICUs provoked moderate or tolerable levels of psychological fatigue in both nurses and nursing technicians. Studies based on psychodynamics corroborate the need for adequate care of workers' mental health [27, 28], given the constant exposure to strenuous and negative experiences arising from the suffering and death of patients [27].

The witnessing of suffering in weak and dependent patients generates a wide range of contradictory feelings, such as pity, compassion, and particularly a sensation of helplessness, all of which are difficult to deal with in a daily work setting, potentially generating psychosomatic disturbances [27].

Dealing with death and dying is another strong source of suffering for nurses and nursing technicians. Western culture does not adequately address these issues—nor are they frequently discussed at health institutions, especially hospitals, which are considered places of healing [1].

Mental hazards are also provoked among nurses and nursing technicians due to responsibility burdens, and the need for precision in carrying out their tasks, since the slightest fault may injure, leave sequels, or even cause the death of a patient [1].

In order to tolerate suffering at work, nurses and nursing technicians invariably adopt defensive strategies, such as the fragmentation of patient-caretaker relationships, the depersonalization, disregard, detachment, and denial of personal feelings when providing direct care to patients [26]. These individual defensive strategies may help, but do not reduce, suffering [28].

## 5. Conclusions

In this study, nursing care at ICUs, for both professional categories, did not significantly contribute towards the occurrence of social hazards, in spite of the need to work night shifts and on weekends, commonly considered burdensome.

In conclusion, the present study suggests that there was no significant difference with regard to physical, psychological, and social hazards between the beginning and end of the careers of ICU nurses and nursing technicians, demonstrating that there is no cumulative effect, since fatigue is produced by the work itself. A plausible explanation may be the adaptation of workers to the existing working conditions.

It must be pointed out that one of the limitations of this study was the insufficient development of the subjective dimensions related to the physical, psychological, and social hazards faced by ICU nurses and nursing technicians. A second limitation is the possible presence of bias, common to transversal studies. If larger samples of workers were studied, other events such as sick leaves or even retirement would also be included as factors associated with suffering at work. These phenomena have yet to be detected by transversal studies. The third limitation is related to the sampling bias due to the study locus, that is, one single institution. Results may not reflect the situation of the general population of ICU nurse and nurse technicians, not only in Brazil, but in other countries. Recall bias may also be present when referring to the beginning of career.

In spite of being collected by a single interviewer, bias was also a concern with regard to the data, since socially desirable answers may have been provided by interviewees, as well as variables subject to manipulation.

With regard to further research, this study indicates the need to develop an agenda aimed at investigating work processes in more detail, (work post and activity analysis), the underlying contradictions between pleasure and suffering, and the role played by mediation strategies in relation to health and changing working conditions, which may be addressed through the continuous education of workers engaged in the above-mentioned activities.

## Figures and Tables

**Table 1:** Medians, means, and standard deviations of physical, psychological, and social hazard factors among nurses and nursing technicians, Brasília, 2007.

Hazard factors	Nurses				Nursing technicians				Total			
	*N*	Md	Ma	SD	*N*	Md	Ma	SD	*N*	Md	Ma	SD
Physical factors	26	2.8	2.7	0.7	96	2.5	2.6	0.8	122	2.6	2.6	0.8
Psychological factors	26	1.7	1.8	0.6	96	1.7	1.8	0.8	122	1.7	1.8	0.8
Social factors	26	1.5	1.7	0.7	96	1.4	1.7	0.7	122	1.4	1.7	0.7

*n*: absolute frequency, Ma: mean; Md: median; SD: standard deviation.

**Table 2:** Medians, means, and standard deviations of physical damage among nurses and nursing technicians, Brasília, 2007.

Physical hazards	Nurses			Nursing technicians		
	Md	Ma	SD	Md	Ma	SD
Sleep disturbances	4.5	3.8	1.4	3	3	1.3
Leg pains	4	3.8	1	4	3.7	1.2
Back pains	3	3.2	1.2	4	3.3	1.3
Body pains	3	3.1	1.1	3	3	1.2
Circulatory disturbances	3	2.6	1.3	2	2.6	1.5
Arm pains	2	2.3	0.9	3	2.8	1.3
Shifts in appetite	2	2	1	2	2.1	1.3
Digestive disturbances	2	2.5	1.3	2	2	1.1
Visual disturbances	1	1.8	1.1	2	2.1	1.2
Auditory disturbances	1	1.5	0.7	1	1.7	1.1
Respiratory disturbances	1	1.9	1.2	1	1.5	1

Ma: mean; Md: median; SD: standard deviation.

**Table 3:** Medians, means, and standard deviations of psychological damage among nurses and nursing technicians, Brasília, 2007.

Psychological hazards	Nurses			Nursing technicians		
	Md	Ma	SD	Md	Ma	SD
Sadness	3	2.5	0.9	2	2.3	1.2
Irritation with everything	2	2.5	1.2	2	2.2	1.1
Loss of self-confidence	2	2	1	1	1.7	0.8
Feeling of emptiness	2	1.7	0.9	2	1.9	1.2
Loss of self-control	1.5	1.8	1	1	1.5	0.9
Bitterness	1.5	1.6	0.8	1	1.7	1
Feeling of defeat	1	1.6	0.9	1	1.5	0.9
Crying for no apparent reason	1	1.5	0.9	1	1.7	1
Willingness to give everything up	1	1.5	0.9	1	1.6	0.9
Long-lasting feeling of despair	1	1.4	0.7	1	1.8	1.1
Negative image of oneself	1	1.4	0.8	1	1.5	0.9

Ma: mean; Md: median; SD: standard deviation.

**Table 4:** Medians, means, and standard deviations of social damage among nurses and nursing technicians, Brasília, 2007.

Social hazards	Nurses			Nursing technicians		
	Md	Ma	SD	Md	Ma	SD
Family relation difficulties	2	2	1.1	1	1.7	1
Affective relation difficulties	1.5	1.7	0.9	1	1.8	1.1
Insensitivity towards others	1	1.7	0.9	1	1.7	1
Social life difficulties	1	1.6	0.8	1	1.6	0.9
Find it difficult to make friends	1	1.6	0.8	1	1.6	0.9
Social isolation	1	1.5	0.9	1	1.6	1
Difficulty in making decisions regarding personal life	1	1.5	0.9	1	1.6	0.9
Overall disinterest towards others	1	1.5	0.8	1	1.7	0.9
Uncontrolled aggressiveness	1	1.3	0.5	1	1.3	0.7

Ma: mean; Md: median; SD: standard deviation.

**Table 5:** Physical, psychological, and social hazards among nurses and nursing technicians at the beginning and end of their careers, according to the Kruskal Wallis test, Brasília, 2007.

Type of hazard	Nurses (*ranks*)				Nursing technicians (*ranks*)			
	*N*	Beginning of career	End of career	*P* values	*N*	Beginning of career	End of career	*P* values
Social hazard	21	12.1	8.7	.23	73	36.8	37.2	.15
Psychological hazard	21	12	8.9	.20	73	39	32.9	.34
Physical hazard	21	11.2	10.5	.15	73	37.3	36.2	.13
